# CC Chemokine Ligand-2: A Promising Target for Overcoming Anticancer Drug Resistance

**DOI:** 10.3390/cancers14174251

**Published:** 2022-08-31

**Authors:** Zhenbo Shi, Jian Tu, Ying Ying, Yunlian Diao, Ping Zhang, Shu Liao, Zhijuan Xiong, Shibo Huang

**Affiliations:** 1 The Clinical Trial Research Center, The First Affiliated Hospital of Nanchang University, Nanchang 330006, China; 2 Jiangxi Province Key Laboratory of Tumor Pathogens and Molecular Pathology, Department of Pathophysiology, School of Basic Medical Sciences, Nanchang University Medical College, Nanchang 330006, China; 3 Jiangxi Medical Center for Major Public Health Events, The First Affiliated Hospital of Nanchang University, Nanchang 330013, China

**Keywords:** cancer, CCL2, drug resistance, TME, therapeutic target

## Abstract

**Simple Summary:**

Drug resistance is an obstacle to cancer therapy, and the underlying mechanisms are still being explored. CC chemokine ligand-2 (CCL2) is one of the key proinflammatory chemokines that regulate the migration and infiltration of multiple inflammatory cells, such as monocytes and macrophages. CCL2 can be secreted by tumor cells and multiple cell types, mediating the formation of the tumor-promoting and immunosuppressive microenvironment to promote cancer development, progression, and anticancer drug resistance. Notably, CCL2 is also frequently overexpressed in drug-resistant cancer cells. Here, we review recent findings regarding the role of CCL2 in the development of resistance to multiple anticancer reagents. In addition, the possible mechanisms by which CCL2 participates in anticancer drug resistance are discussed, which may provide new therapeutic targets for reversing cancer resistance.

**Abstract:**

CC chemokine ligand-2 (CCL2), a proinflammatory chemokine that mediates chemotaxis of multiple immune cells, plays a crucial role in the tumor microenvironment (TME) and promotes tumorigenesis and development. Recently, accumulating evidence has indicated that CCL2 contributes to the development of drug resistance to a broad spectrum of anticancer agents, including chemotherapy, hormone therapy, targeted therapy, and immunotherapy. It has been reported that CCL2 can reduce tumor sensitivity to drugs by inhibiting drug-induced apoptosis, antiangiogenesis, and antitumor immunity. In this review, we mainly focus on elucidating the relationship between CCL2 and resistance as well as the underlying mechanisms. A comprehensive understanding of the role and mechanism of CCL2 in anticancer drug resistance may provide new therapeutic targets for reversing cancer resistance.

## 1. Introduction

Drug resistance, including natural resistance and acquired resistance, is still an obstacle to cancer therapy [1]. Several mechanisms, such as the overexpression of ATP binding cassette (ABC) transporters, mutations in drug targets, DNA damage repair [2], and regulation of cell apoptosis [3], contribute to the development of drug resistance. Recently, the role of chemokines in the development of resistance has attracted much interest [4,5].

Chemokines are a family of small-molecule cytokines that interact with their receptors to mediate chronic inflammation and an immunosuppressive tumor microenvironment (TME), facilitating tumor development, tumor progression, and drug resistance [6]. Accumulating studies, including in vitro, in vivo, and clinical studies, demonstrated that several chemokines were significantly upregulated in resistant cell lines or patients [7]. Furthermore, these upregulated chemokines mediate drug resistance through the recruitment of immune cells [8], activation of the survival/proliferation pathway [9], and promotion of invasion [10]. A variety of chemokines have been reported to be associated with cancer resistance, such as C-X-C chemokine ligand-8 (CXCL8) [11], CC chemokine ligand-5 (CCL5) [12], CC chemokine ligand-20 (CCL20) [8], and CC chemokine ligand-2 (CCL2). For instance, CXCL8 has been reported to induce chemoresistance through the ABCB1 pathway, and its inhibition could re-sensitize resistant cells to doxorubicin [11]. Blocking CCL20 prevented tumor progression and restored 5-FU sensitivity in colorectal cancer [8]. Collectively, chemokines may be promising potential therapeutic targets to improve anticancer drug efficacy.

**Figure 1:**
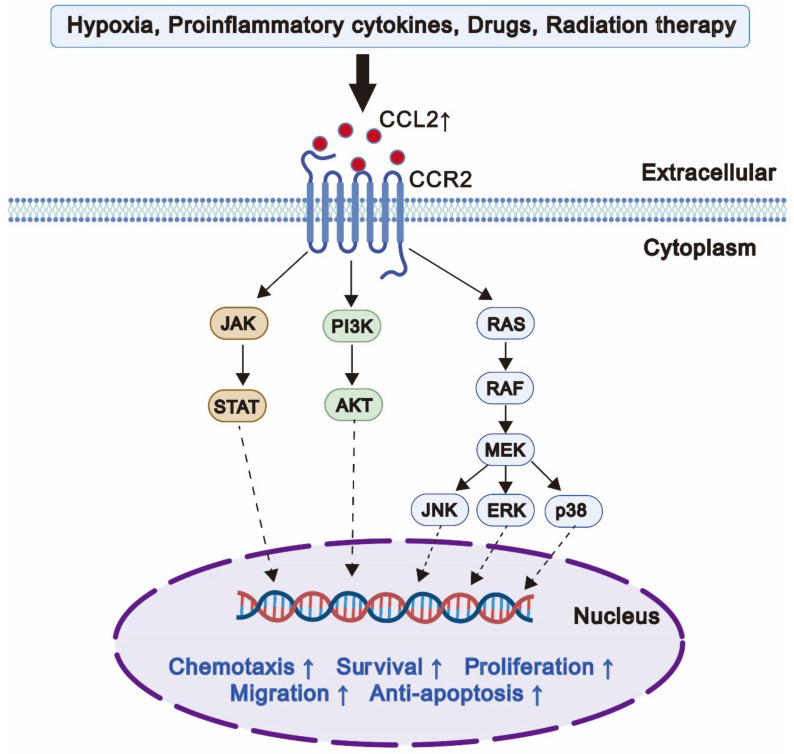
The signaling pathways and multiple biological processes of CCL2. Multiple factors such as hypoxia, proinflammatory cytokines, drugs, and radiation therapy increase the production of CCL2. After binding to CCR2, various intracellular G protein-mediated downstream signaling pathways are activated, such as JAK/STAT, PI3K/AKT, and MAPK pathways, which are related to chemotaxis, survival, proliferation, migration, and anti-apoptosis.

CCL2, also known as monocyte chemotaxis protein-1 (MCP-1), is one of the first identified chemokines [13]. The CCL2 gene is located in human chromosome 17 (chr.17.0 q11.2), which expresses a 13 kDa protein composed of 76 amino acid residues [14]. CCL2 is mainly secreted by macrophages and tumor cells, and it can also be secreted by fibroblasts [15], endothelial cells [16], and dendritic cells [17]. CCL2 has been identified to be a potent chemokine for inflammation, which recruits many kinds of immune cells, such as macrophages [18], T lymphocytes [19], natural killer cells [20], and neutrophils [21]. In particular, the differentiation and survival of tumor-associated macrophages (TAMs) depend on CCL2. CCL2 can exert its biological effects by recruiting these multiple types of cells. The major receptor of CCL2 is CCR2 [22]. Upon binding of CCL2 to the N-terminus of CCR2, various intracellular G protein-mediated downstream signaling pathways are activated, including the JAK-STAT pathway, MAPK pathway, and PI3K-AKT pathway, which regulate multiple biological processes, including chemotaxis, cell survival, proliferation, migration, and anti-apoptosis (Figure 1). Furthermore, CCL2 plays a particularly important role in cancer progression. CCL2 can be secreted into the TME, serving as one of the crucial mediators of complex interactions between tumor and host cells [23], which promote tumor proliferation [24], angiogenesis [25], and metastasis [26] and reduce the response to therapy by facilitating the formation of a tumor-promoting and immunosuppressive tumor microenvironment.

Recently, extensive research has focused on the role of CCL2 in the development of resistance to cancer therapy [27,28,29,30]. It has been reported that CCL2 expression is often higher in drug-resistant tumor cell lines than in drug-sensitive tumor cell lines, and silencing or blockade of its expression can re-sensitize cancer cells to anticancer treatment [27,28,29,30]. Accumulating studies suggest that CCL2 is associated with resistance to chemotherapy [7,31,32], targeted therapy [29], hormonal therapy [28], and immunotherapy [30] (Table 1). However, an integrated understanding of the association of CCL2 with anticancer drug resistance is lacking. In this review, we discuss and summarize the findings regarding the role of CCL2 in the development of resistance to multiple drugs. Additionally, we focus on the potential mechanisms by which CCL2 participates in the process of anticancer drug resistance. Such insights could present us with an option for designing more effective therapies to overcome cancer resistance.

## 2. CCL2 Induces Resistance to Chemotherapy

### 2.1. Resistance to Taxanes

Taxanes, including paclitaxel, docetaxel, and cabazitaxel, are a type of first-line chemotherapy agent that inhibits cell growth by stopping mitosis. It has been demonstrated that CCL2 may be a taxane-resistance-associated protein [7,31,32,33,34].

A clinical study found that circulating CCL2 was remarkably elevated in patients who had no response to platinum- and taxane-based chemotherapy [31]. Similarly, the upregulation of CCL2 has been reported in docetaxel and cabazitaxel-resistant cancer cells [32,33,34]. CCL2 was significantly upregulated when treated with docetaxel combined with mitoxantrone. Furthermore, it has been shown that just docetaxel, not mitoxantrone, induced CCL2 expression in a dose-dependent manner in LNCaP and LAPC4 prostate cancer cell lines [33]. Conversely, increasing CCL2 reduces the efficacy of docetaxel [34]. Furthermore, knocking down CCL2 can inhibit cell proliferation and enhance the effect of docetaxel.

Additionally, the results of cDNA microarray analysis showed that CCL2 was upregulated in cabazitaxel-resistant cells compared to the cabazitaxel-sensitive cells, and CCL2 was the most upregulated in the 24-CCL subfamily (CCL1, CCL28, etc.) [32]. Furthermore, blocking the CCL2-CCR2 axis could increase caspase-3 and PARP levels and restore the sensitivity of cabazitaxel-resistant cells to cabazitaxel. The author also constructed a mouse model and demonstrated that a combination of CCR2 antagonists with cabazitaxel was more effective at inhibiting tumor growth than cabazitaxel and CCR2 antagonists alone. CCL2 blockade could enhance the efficacy of paclitaxel and carboplatin [35]. These findings suggest that CCL2 may contribute to taxane chemoresistance.

### 2.2. Resistance to Platinum Drugs

Platinum complexes are a class of anticancer drugs that disrupt DNA structure and function [47]. Three compounds have been approved by the FDA: cisplatin, carboplatin, and oxaliplatin [48]. Studies have shown that CCL2 is associated with platinum resistance [27,36]. For instance, Duranyildiz et al. [31] reported increased CCL2 levels in the sera of patients resistant to platinum- and taxane-based therapies. Studies demonstrated that cisplatin-resistant cancer cells secrete more CCL2 than cisplatin-sensitive cancer cells, and knockdown of CCL2 successfully reverses cisplatin resistance. Furthermore, cisplatin-sensitive cancer cells can acquire resistance to cisplatin after being cocultured with cisplatin-resistant cells [27]. These findings indicate that CCL2 mediates resistance to platinum drugs.

### 2.3. Resistance to Temozolomide

A very recent study demonstrated that CCL2 promotes temozolomide (TMZ) resistance in glioma cells [37]. The study found that CCL2 was significantly upregulated in TMZ-resistant glioma cells compared to the parental cell lines. Further in vivo study confirmed that overexpression of CCL2 significantly reduced the antitumor effect of TMZ, indicating that overexpression of CCL2 is the cause of TMZ resistance. Moreover, the study demonstrated that CCL2 induced resistance to temozolomide by reducing TMZ-induced apoptosis by the activation of the AKT pathway and the promotion of glycolysis.

## 3. CCL2 Reduces the Sensitivity of Cancer to Hormone Therapy

Hormone therapy mainly changes the disordered state of hormone balance through hormones or its antagonist drugs, which suppress the growth of hormone-dependent cancer. There is evidence that CCL2 may lead to resistance to hormone therapy by facilitating tumor metastasis and suppressing apoptosis [38,39].

Tamoxifen is frequently used for the treatment of estrogen receptor-positive breast cancer without apparent adverse effects [49]. A recent study revealed that TAMs promote the development of tamoxifen resistance by secreting CCL2 [38]. Firstly, they found that tamoxifen-resistant breast cancer cells significantly induced more macrophage M2 polarization compared to tamoxifen-sensitive cells. In turn, the conditioned media of M2-polarized macrophages induced breast cancer cells resistant to tamoxifen [38]. Then, they analyzed the expression of seven cytokines, including CXCL-1, CCL2, CCL5, IL-6, IL-8, IL-17, and CXCL-5, to investigate the possible mechanism, and they found that only the expression of CCL2 was statistically increased. Mechanistically, CCL2 could inhibit apoptosis and mediate tamoxifen resistance by activating the PI3K/Akt/mTOR signaling pathway. Additionally, CCL2 recruits monocytes to the TME, thereby promoting the formation of the tamoxifen resistance microenvironment. Furthermore, after endocrine therapy, the progression-free survival rate in patients with high CCL2 expression in the stroma was shorter than in those with low CCL2 expression [38]. Altogether, these results suggest that CCL2 leads to tamoxifen resistance and can serve as a novel therapeutic target to overcome tamoxifen resistance in breast cancer patients.

As androgen receptor (AR) signaling is important for the development of prostate cancer, androgen deprivation therapy (ADT) is a first-line treatment for metastatic prostate cancer (PCa) [50]. Although ADT is initially effective in the majority of patients, CRPC (castration-resistant prostate cancer) will develop in advanced prostate cancer [51]. Currently, CCL2 has been confirmed to be associated with the induction of CRPC and may be a promising target for preventing castration resistance [28,39]. Izumi et al. [39] elucidated that AR silencing could induce increased CCL2 expression, which in turn recruited TAM and also enhanced PCa metastasis. Further in vitro and in vivo studies demonstrated that blockade of the CCL2/CCR2-STAT3 axis could inhibit PCa metastasis. Additionally, they analyzed the expression changes of CCL2 in four patients with CRPC to investigate whether CCL2 contributes to the development of CRPC. Intriguingly, they found that CCL2 expression increased after ADT-sensitive patients developed CRPC, and PCa patients with increased CCL2 expression had a poor prognosis. These findings indicated that CCL2 promotes the development of PCa progression, but its linkage to the development of CRPC needs further investigation.

Recently, Lee et al. [28] revealed that overexpression of WNT5A results in LNCaP cells becoming resistant to castration and causes increased expression of CCL2, which can recruit TAMs to tumor sites. Notably, an in vivo study showed that the removal of macrophages significantly attenuated WNT5A-induced CRPC, indicating that WNT5A induces CRPC by upregulating the expression of CCL2. Similarly, a recent study demonstrated that CCL2 is involved in the development of FOXA1 loss-induced CRPC progression [52]. These results indicate that a combination of targeting AR and anti-CCL2/CCR2 may be a better therapeutic strategy to prevent PCa progression at the castration-resistant stage.

## 4. CCL2 Leads to the Development of Resistance to Targeted Therapy

Targeted therapy plays an anticancer role mainly by interfering with key targets related to tumor development. Due to the advantages of mild side effects and confirmed efficacy, targeted therapy has been increasingly widely used in clinics in recent years. Recent studies have revealed that CCL2 reduces the effectiveness of multiple targeted therapies, such as bevacizumab, sorafenib, vemurafenib, and trastuzumab, which are mainly related to CCL2-mediated angiogenesis and anti-apoptosis.

### 4.1. Resistance to Anti-VEGF Drugs

The vascular endothelial growth factor (VEGF) family and its receptors are essential for angiogenesis, which plays an important role in cancer progression. A study found that VEGF is overexpressed in tumors and is considered a valid biomarker of the tumor [53]. Therefore, agents that directly or indirectly target VEGF have been developed for cancer therapy.

Bevacizumab, a humanized VEGF-A-targeting monoclonal antibody, has been approved for the treatment of metastatic colorectal cancer, metastatic breast cancer, non-small-cell lung cancer, glioblastoma, renal cell carcinoma, ovarian cancer, and cervical cancer [54]. Recently, a study found that colorectal cancer patients with positive expression of ETV5, a member of the ETS transcription factor family that can trigger angiogenesis by upregulating VEGFA, are resistant to bevacizumab [29]. An in-depth mechanistic study found that ETV5 not only upregulated VEGFA but also increased the expression of CCL2 [29]. Additionally, these factors are two parallel signals that sustain angiogenesis for tumor development. During bevacizumab treatment of colorectal cancer patients with positive expression of ETV5, the secretion of CCL2 induced by ETV5 resulted in persistent angiogenesis, indicating that CCL2 plays a critical role in bevacizumab resistance. Indeed, the combination of anti-CCL2 and bevacizumab inhibited ETV5-positive colorectal cancer angiogenesis more efficiently than either of them alone [29]. Similarly, a recent study of glioblastoma confirmed that CCL2 expression can enhance resistance to bevacizumab by recruiting macrophages [40].

Sorafenib, a multi-kinase inhibitor that inhibits Raf, PDGFR, and VEGFR signaling, has been widely used in the treatment of liver cancer [55]. Zhou et al. indicated that, following sorafenib treatment, more tumor-associated neutrophils (TANs), macrophages, and Treg cells infiltrated into the tumor [41]. They also found that decreased TANs significantly enhanced the sensitivity to sorafenib in a mouse model [41]. This suggests that TANs promote sorafenib resistance in HCC cells. Furthermore, TANs highly expressed CCL2 and CCL17 through the PI3K/Akt and p38/MAPK signaling pathways, which promoted intratumor infiltration of macrophages and Treg cells. Notably, the number of CCL2+ or CCL17+ TANs was associated with the microvascular invasion, size, differentiation, and stage of the tumor [41]. Inhibition of CCL2 or its receptor significantly reduced the migration of macrophages and Treg cells. These findings indicate that CCL2 plays an important role in sorafenib resistance.

### 4.2. Resistance to BRAF Inhibitors

Accumulating studies demonstrate that a BRAF inhibitor (BRAFi) can significantly improve the survival of cancer patients with BRAF mutations [56,57]. Recent studies have shown that CCL2 is related to the development of resistance to BRAFi. Steinberg et al. [42] found that BRAFi initially decreases the expression of CCL2, but long-term treatment with BRAFi causes CCL2 to increase above its initial level. Additionally, Vergani et al. [43] revealed that cancer cells that are resistant to BRAFi (vemurafenib) produce increased CCL2, which, in turn, promotes the development of resistance. Downregulation of CCL2 restored apoptosis in resistant cells and improved vemurafenib efficacy.

Notably, it has been demonstrated that CCL2-mediated acquired resistance to BRAFi may be related to myeloid-derived suppressor cells (MDSCs) [42]. CCL2 secreted by melanoma cells is a key mediator of MDSC recruitment to tumors. Although blocking CCR2 alone was not sufficient to inhibit tumor growth, resistance to BRAFi was abolished when CCR2 antagonists were used in combination with other drugs (anti-CTLA-4 and anti-PD-1 drugs). These studies confirm that targeting CCL2 is a promising strategy to reverse BRAF inhibitor resistance.

### 4.3. Resistance to Trastuzumab

Trastuzumab has been widely used in HER2+ patients with breast carcinoma. Recent studies suggest that CCL2 affects trastuzumab efficacy in breast cancer [58] and induces trastuzumab resistance in HER2+ gastric cancer [44]. Tagliabue et al. treated FVB mice that bore HER2+ mammary carcinoma cells (MI6) with trastuzumab with or without a CCL2-blocking monoclonal antibody and found that treatment with anti-CCL2 significantly enhanced the activities of trastuzumab [58], indicating that the therapeutic activity of trastuzumab is dependent on the CCL2 level. Fu et al. [44] demonstrated that HER2+ gastric cancer patients with higher CCL2 expression had a poorer prognosis. Moreover, patients with innate resistance to trastuzumab had significantly higher expression of CCL2. Notably, they established a stable CCL2-overexpressing HER2+ gastric cancer cell line and found that tumor cell-derived CCL2 had no direct effect on trastuzumab resistance. However, CCL2 overexpression remarkably reduced the inhibitory activity of trastuzumab when coculturing the CCL2-overexpressing cell lines with induced TAMs. An in vivo study showed that CCL2-overexpressing cell lines were resistant to trastuzumab. CD40 and HER2 bispecific antibodies, which specifically target the HER2 and CD40 signaling pathways, significantly upregulated the M1-like phenotype of TAMs and overcame trastuzumab resistance. These findings suggest that CCL2 can elicit trastuzumab resistance via regulation of TAMs.

## 5. CCL2 Leads to Resistance to Immunotherapy

Cancer immunotherapy effectively inhibits cancer by enhancing the immunogenicity of tumor cells and the sensitivity to the killing of effector cells. Although immunotherapy is often more effective than conventional chemotherapy and targeted therapies, many patients still develop resistance to immunotherapy [59]. PD-1/PD-L1 inhibitors, which enhance T cell immunity to impede tumor immune evasion, are considered one of the most successful cancer immunotherapy approaches [60] used in a variety of cancer treatments, such as non-small cell lung cancer (NSCLC) [61], Hodgkin’s lymphoma [62], and melanoma [63]. However, many factors can contribute to resistance to PD-1/PD-L1 inhibitors, such as the presence of STK11 and KEAP1 mutations [64].

Recently, CCL2 has been confirmed to be associated with PD-1/PD-L1 inhibitor resistance [30,46]. The study demonstrated that increased CCL2 expression, which is induced by the activation of PI3K/AKT and NF-κB signaling, is the cause of intrinsic PD-1/PD-L1 inhibitor resistance [30]. The authors found that activation of PI3K/AKT and NF-κB induced the upregulation of both PD-L1 and CCL2 in breast cancer cells. Interestingly, the expression of CCL2 was still increased despite blocking PD-L1 by a PD-L1 inhibitor. Dual blockade of CCL2 and PDL1 by siRNA and the PD-L1 inhibitor induced a higher level of T cell-mediated apoptosis than siCCL2 and PD-L1 inhibitor alone [45].

Similarly, Wang et al. proved that environmental eustress could overcome PD-L1/PD-1 resistance by silencing CCL2 [46]. The EE (enriched environment) is known to be an environmental eustress model that can reduce mouse anxiety and make mice happier [46]. The researchers found that EE downregulated CCL2 expression in tumor cells and tumor-associated immune cells through the peripheral neuroendocrine–immune pathway sympathetic nervous system (SNS)/β-adrenergic receptors (β-ARs)/CCL2. Accordingly, downregulation of CCL2 expression enhanced CD8+ T cell-mediated antitumor immunity, which mitigates cancer immunosuppression and overcomes PD-1 immunotherapy resistance by inhibiting TAM and G-MDSC infiltration.

## 6. Putative Mechanisms of CCL2-Mediated Drug Resistance in Cancer

### 6.1. CCL2 Induces Resistance via Inhibition of Apoptosis and Autophagy

**Figure 2:**
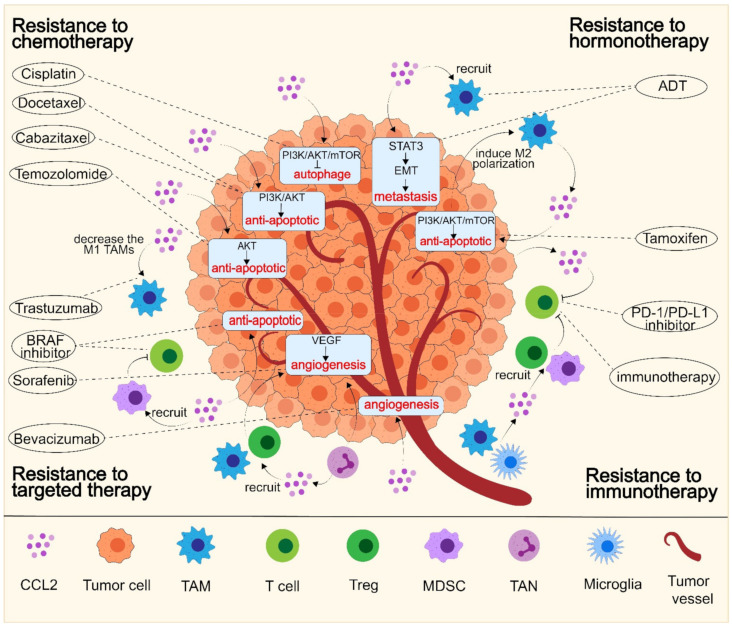
The role and mechanism of CCL2 in the development of anticancer resistance. CCL2 expression is increased in multiple resistant cancer cells and can interact with multiple cells in the TME, thereby leading to resistance to chemotherapy, hormone therapy, targeted therapy, and immunotherapy by: (1) suppressing apoptosis and autophagy; (2) facilitating tumor metastasis through EMT; (3) increasing angiogenesis; and (4) forming an immunosuppressive TME.

A previous study indicated that chemokines and growth factors produced by a tumor could promote proliferative and antiapoptotic signals, helping the tumor to escape drug-mediated destruction [65]. Similarly, recent studies have revealed that CCL2 could activate the PI3K/AKT pathway and resist tumor cell apoptosis and autophagy, thereby leading to resistance to major anticancer agents, such as taxanes [33,34], platinum drugs [27], tamoxifen [38], and temozolomide [37] (Figure 2).

Qian et al. [33] found that overexpressed CCL2 could activate the PI3K/AKT signaling pathway to inhibit apoptosis-related protein caspase-3 activation and Bcl-2 phosphorylation. Similarly, Natsagdorj et al. [32] indicated that cabazitaxel could induce apoptosis by activating caspase-3 and poly ADP-ribose polymerase (PARP). However, overexpression of recombinant human CCL2 (rhCCL2) decreased the apoptotic rate induced by cabazitaxel through inactivation of caspase-3 and PARP. Furthermore, blocking the CCL2-CCR2 axis could increase caspase-3 and PARP levels and restore the sensitivity of cabazitaxel-resistant cells to cabazitaxel. Additionally, CCL2 mediated tamoxifen resistance by similar mechanisms.

Autophagy is widely believed to promote tumor survival. However, activation of autophagy has also been reported to improve chemotherapy sensitivity [66]. Xu et al. found that CCL2 induces cisplatin resistance in gastric cancer cells by inhibiting autophagy [27]. Mechanistically, CCL2 attenuated cytotoxicity induced by cisplatin by activating PI3K-Akt-mTOR signaling to inhibit autophagy. Knockdown of CCL2 or autophagy induction successfully reversed cisplatin resistance. In addition, CCL2 also increased the expression of SQSTM1, one of the autophagic receptors that are degraded by autophagy in autolysosomes [67]. In turn, SQSTM1 activated CCL2 transcription via the NF-κB signaling pathway, representing a positive feedback loop to sustain drug resistance [27].

### 6.2. CCL2 Leads to Resistance by Promoting Angiogenesis

Tumor angiogenesis is an indispensable process for tumorigenesis and development. Multiple cytokines are known to be involved in angiogenesis, such as VEGF, GM-CSF, and FGFs. CCL2 also accelerates tumor development by increasing angiogenesis in several kinds of cancers, such as hepatocellular carcinoma [68], prostate cancer, esophageal squamous carcinoma, and gastric carcinoma [25] (Figure 2). It has been reported that CCL2 mediates tumor angiogenesis in two main ways: CCL2 can bind to CCR2 directly, leading to angiogenesis [69,70], and CCL2 may indirectly promote angiogenesis through the recruitment of macrophages [25,71]. VEGF is also involved in CCL2-mediated angiogenesis. In PC-3M prostate cancer cells, CCL2 was found to indirectly cause angiogenesis by upregulating VEGF expression in tumor cells [72].

In cancer treatment, antiangiogenic drugs inhibit important proangiogenic factors, but CCL2, as another key proangiogenic factor, neutralizes the effect of antiangiogenic drugs. Recently, it has been reported that CCL2 induced anticancer agent resistance by promoting neovascularization formation, leading to resistance to temozolomide, bevacizumab, and sorafenib [73]. Feng et al. [29] indicated that, while bevacizumab targets VEGFA to inhibit tumor angiogenesis, ETV5-mediated CCL2 secretion acts as a parallel bypass to increase tumor angiogenesis to promote bevacizumab resistance. Additionally, Zhou et al. indicated that sorafenib treatment induces increased expression of CCL2 and infiltration of neutrophils, macrophages, and Treg cells, which promote angiogenic function through VEGF [74,75] to neutralize the antiangiogenic effect of sorafenib.

### 6.3. CCL2 Mediates Resistance by Facilitating Tumor Metastasis

Tumor metastasis is one of the major causes of death in cancer patients. The vital role of CCL2 in facilitating tumor metastasis has been demonstrated in diverse cancers, which involve the activation of multiple signaling pathways, including the MAPK, JAK/STAT, and PI3K/AKT pathways. Many studies indicate that CCL2 facilitates tumor cell metastasis by promoting epithelial-to-mesenchymal transformation and the recruitment of macrophages [26,76] (Figure 2).

ADT with antiandrogens could lead to a reduction in primary tumors yet may lead to increased metastasis in some PCa patients [77]. CCL2 may contribute to ADT-induced PCa metastasis. It has been reported that the inhibition of AR signaling promotes CCL2 secretion in prostate cancer cells. In turn, the upregulated CCL2 promotes STAT3 phosphorylation, leading to enhanced epithelial–mesenchymal transition (EMT) and metastasis of prostate cancer cells, thus promoting prostate cancer cell castration resistance [39]. Similarly, during the development of bicalutamide resistance, CCL2 induces increased migration and invasion of prostate cancer cells by promoting AKT phosphorylation [78].

### 6.4. CCL2 Mediates Resistance by Forming an Immunosuppressive TME

The TME consists of a variety of cellular and non-cellular elements, such as inflammatory cells, immune cells, bone marrow-derived cells, cancer-associated fibroblasts, micro-vessels, extracellular matrix, and a variety of growth factors as well as chemokines [79]. It has been indicated that the TME has a significant effect on cancer progression and serves as an important target for cancer therapy [79]. CCL2, as an important medium between the tumor and TME, can be secreted by multiple cells in the TME and interacts with immune cells infiltrated in the TME [23] (Figure 2). Normally, immune cells function to eliminate malignancies that are harmful to the host, whereas some immune cells in the TME promote tumor cell growth and metastasis [80]. Furthermore, in recent years, accumulating studies have suggested that the TME affects the sensitivity of cancer to drugs [4,5]. In this study, we focused on several immune cells in the TME as well as their interactions with CCL2.

Macrophages are one of the leukocytes in the natural immune system, which can be polarized into two different types: M1 and M2 [81]. In contrast to M1, M2 macrophages inhibit the immune response and promote tumor progression [82]. Moreover, tumor-associated macrophages (TAMs) clustered in the tumor microenvironment are considered a particular phenotype of M2 macrophages [82], because their function is similar to that of M2 macrophages [83]. It was established that TAMs play an important role in CCL2-mediated tumorigenesis and development. CCL2 promotes tumor progression by recruiting TAMs [40], but TAMs also induce cancer resistance by secreting CCL2 [38]. This interaction facilitates tumor growth and metastasis in the TME.

Neutrophils are involved in various immune processes, such as inflammation, autoimmunity, and cancer [84]. Tumor-associated neutrophils (TANs) secrete CCL2 to facilitate the progression of cancer. It was reported that CCL2 secreted by TANs promoted lymph node metastasis from oral squamous cell carcinoma (OSCC) [85]. Consistently, melatonin suppressed CCL2 secreted by TANs via blockage of P38/MAPK and AKT, retarding OSCC metastasis [86]. In addition, CCL2 could also induce TANs to express PD-L1, which inhibited tumor immunity in hepatocellular carcinoma [87].

Regulatory T cells (Tregs) refer to CD4+ CD25+ Foxp3+ T cells. Tregs can promote tumor growth by inhibiting tumor-specific T cell immunity or increasing angiogenesis [75,88]. CCL2-mediated tumorigenesis and development are associated with the Treg recruitment process. CCL2 can lead to the activation and accumulation of Tregs, thus suppressing antitumor immunity in breast cancer tissues [89]. However, downregulation of cancer cell-derived CCL2 inhibited the interaction between breast cancer cells and Tregs, thus inhibiting the proliferation and migration of breast cancer cells [90]. Moreover, expression of CCL2 induced by radiotherapy mediates the recruitment of CCR2+ Tregs in a mouse model of head and neck squamous cell carcinoma, thus diminishing the efficacy of radiotherapy, indicating that CCL2 may be a potential target to improve the efficacy of radiotherapy [91].

MDSCs are a group of immature immunosuppressive cells from the bone marrow whose primary function is to inhibit the T cell response [92]. CCL2 contributes to the progression of cancer via recruiting MDSCs to form a tumor-supportive immune microenvironment, whereas the inhibition of CCL2 production diminished both the accumulation of MDSC and tumor growth [93]. In CCL2-null mice, the number of MDSCs is decreased, but CD8+ T cells are increased, which attenuates the progression of BRAFi-resistant gliomas [94].

## 7. Conclusions

In this review, we summarize the roles and potential mechanisms of CCL2 in the development of anticancer drug resistance. However, there are still gaps in our understanding of the processes by which CCL2 contributes to the development of anticancer drug resistance. Firstly, some studies lack direct and solid scientific evidence supporting that CCL2 plays causative roles in inducing resistance [95]. For instance, many studies just observed that CCL2 was upregulated in resistant cancer cells or tissues [95]. In-depth investigation is needed to provide a certain mechanism that CCL2 could affect, such as drug pharmacokinetics, biodistribution, or action. Secondly, the mechanism by which CCL2 induces resistance is not fully understood. For example, although CCL2 secreted by M2-polarized TAMs could activate the PI3K/Akt/mTOR pathway [38], the direct mechanism underlying the CCL2-induced activation of this pathway has not yet been elucidated. Finally, the regulatory mechanism of CCL2 secretion remains unclear, as it may vary based on different cellular contexts or drug treatments. For example, the mechanism of inducing CCL2 expression is different for gefitinib [95] compared to erlotinib [96].

At present, CCL2-targeted therapy or its combination with other drugs has been shown to effectively improve the efficacy of antitumor drugs in in vitro experiments and animal models [97,98]. CCL2 or CCR2 antagonists are also undergoing clinical trials [99,100]. However, targeted CCL2 combination therapy is still in an early stage. In conclusion, the mechanisms of CCL2 in inducing drug resistance in cancer are very complicated, and further pre-clinical studies and clinical trials should be considered to investigate whether targeting CCL2 could overcome anticancer drug resistance.

## Figures and Tables

**Table 1:** CCL2 is associated with anticancer drug resistance.

Type of Therapy	Anticancer Drug	Type of Cancer	Resistance Mechanism	Ref.
Chemotherapy	Paclitaxel	Ovarian cancer	-	[7]
	Platinum and taxane	Gastric cancer	-	[31]
	Cabazitaxel	Prostate cancer	Resistance to apoptosis	[32]
	Docetaxel	Prostate cancer	Resistance to apoptosis via PI3K/AKT signaling	[33]
	Docetaxel	Lung cancer	Resistance to apoptosis via PI3K/AKT signaling	[34]
	Paclitaxel and carboplatin	Ovarian cancer	-	[35]
	Cisplatin	Bladder cancer	-	[36]
	Cisplatin	Gastric cancer	Resistance to autophagy via PI3K/AKT/mTOR signaling	[27]
	Temozolomide	Glioma	Resistance to apoptosis via AKT signaling	[37]
Hormone therapy	Tamoxifen	Breast cancer	Resistance to apoptosis via PI3K/AKT/mTOR signaling	[38]
	ADT	Prostate cancer	-	[28]
	ADT	Prostate cancer	Recruit macrophages and promote EMT and metastasis via STAT3 activation	[39]
Targeted therapy	Bevacizumab	Colorectal cancer	Promote angiogenesis	[29]
	Bevacizumab	Glioblastoma	Recruit macrophages and promote angiogenesis	[40]
	Sorafenib	Hepatocellular carcinoma	Recruit macrophages and Treg cells	[41]
	BRAF inhibitor	Melanomas	Recruit MDSCs	[42]
	Vemurafenib	Melanomas	Resistance to apoptosis	[43]
	Trastuzumab	Gastric cancer	Regulate the TAMs phenotype	[44]
Immunotherapy	PD-1/PD-L1 inhibitor	Breast cancer	Inhibit infiltration of cytotoxic T cells	[30]
	Immunotherapy	-	Inhibit infiltration of cytotoxic T cells	[45]
	PD-1/PD-L1 inhibitor	Liver cancer	Inhibit antitumor immunity	[46]

## References

[1] Robey R.W., Pluchino K.M., Hall M.D., Fojo A.T., Bates S.E., Gottesman M.M. (2018). Revisiting the role of ABC transporters in multidrug-resistant cancer. Nat. Rev. Cancer.

[2] Wu S., Li X., Gao F., de Groot J.F., Koul D., Yung W.K.A. (2021). PARP-mediated PARylation of MGMT is critical to promote repair of temozolomide-induced O6-methylguanine DNA damage in glioblastoma. Neuro-Oncology.

[3] Tortosa A., Perez-Tomas R. (2009). Overcoming Drug Resistance by Enhancing Apoptosis of Tumor Cells. Curr. Cancer Drug Targets.

[4] Wu T., Dai Y. (2016). Tumor microenvironment and therapeutic response. Cancer Lett..

[5] Senthebane D.A., Rowe A., Thomford N.E., Shipanga H., Munro D., Al Mazeedi M.A.M., Almazyadi H.A.M., Kallmeyer K., Dandara C., Pepper M.S. (2017). The Role of Tumor Microenvironment in Chemoresistance: To Survive, Keep Your Enemies Closer. Int. J. Mol. Sci..

[6] Ozga A.J., Chow M.T., Luster A.D. (2021). Chemokines and the immune response to cancer. Immunity.

[7] Duan Z., Feller A.J., Penson R.T., Chabner B., Seiden M.V. (1999). Discovery of differentially expressed genes associated with paclitaxel resistance using cDNA array technology: Analysis of interleukin (IL) 6, IL-8, and monocyte chemotactic protein 1 in the paclitaxel-resistant phenotype. Clin. Cancer Res..

[8] Wang D., Yang L., Yu W., Wu Q., Lian J., Li F., Liu S., Li A., He Z., Liu J. (2019). Colorectal cancer cell-derived CCL20 recruits regulatory T cells to promote chemoresistance via FOXO1/CEBPB/NF-κB signaling. J. Immunother. Cancer.

[9] Waldeck S., Rassner M., Keye P., Follo M., Herchenbach D., Endres C., Charlet A., Andrieux G., Salzer U., Boerries M. (2020). CCL5 mediates target-kinase independent resistance to FLT3 inhibitors in FLT3-ITD-positive AML. Mol. Oncol..

[10] Kato T., Fujita Y., Nakane K., Mizutani K., Terazawa R., Ehara H., Kanimoto Y., Kojima T., Nozawa Y., Deguchi T. (2013). CCR1/CCL5 interaction promotes invasion of taxane-resistant PC3 prostate cancer cells by increasing secretion of MMPs 2/9 and by activating ERK and Rac signaling. Cytokine.

[11] Fousek K., Horn L.A., Palena C. (2020). Interleukin-8: A chemokine at the intersection of cancer plasticity, angiogenesis, and immune suppression. Pharmacol. Ther..

[12] Zhang X.-N., Yang K.-D., Chen C., He Z.-C., Wang Q.-H., Feng H., Lv S.-Q., Wang Y., Mao M., Liu Q. (2021). Pericytes augment glioblastoma cell resistance to temozolomide through CCL5-CCR5 paracrine signaling. Cell Res..

[13] Matsushima K., Larsen C.G., Dubois G.C., Oppenheim J.J. (1989). Purification and characterization of a novel monocyte chemotactic and activating factor produced by a human myelomonocytic cell line. J. Exp. Med..

[14] Van Coillie E., Van Damme J., Opdenakker G. (1999). The MCP/eotaxin subfamily of CC chemokines. Cytokine Growth Factor Rev..

[15] Wu S., Kong X., Wang Y., Fan X., Huang Q., Chen R., Yu W., Ma L., Sun Y., Jiang L. (2021). Curcumin alleviates inflammation in Takayasu’s arteritis by blocking CCL2 overexpression in adventitial fibroblasts. Clin. Exp. Rheumatol..

[16] Tumur Z., Shimizu H., Enomoto A., Miyazaki H., Niwa T. (2010). Indoxyl Sulfate Upregulates Expression of ICAM-1 and MCP-1 by Oxidative Stress-Induced NF-ĸB Activation. Am. J. Nephrol..

[17] Novoszel P., Holcmann M., Stulnig G., Fernandes C.D.S., Zyulina V., Borek I., Linder M., Bogusch A., Drobits B., Bauer T. (2021). Psoriatic skin inflammation is promoted by c-Jun/AP-1-dependent CCL2 and IL-23 expression in dendritic cells. EMBO Mol. Med..

[18] Yoshimura T. (2018). The chemokine MCP-1 (CCL2) in the host interaction with cancer: A foe or ally?. Cell. Mol. Immunol..

[19] Cédile O., Wlodarczyk A., Owens T. (2017). CCL 2 recruits T cells into the brain in a CCR 2-independent manner. APMIS.

[20] Lehmann M.H., Torres-Domínguez L.E., Price P.J.R., Brandmüller C., Kirschning C.J., Sutter G. (2016). CCL2 expression is mediated by type I IFN receptor and recruits NK and T cells to the lung during MVA infection. J. Leukoc. Biol..

[21] Wang X.-Z., Zhang S.-Y., Xu Y., Zhang L.-Y., Jiang Z.-Z. (2018). The role of neutrophils in triptolide-induced liver injury. Chin. J. Nat. Med..

[22] Deshmane S.L., Kremlev S., Amini S., Sawaya B.E. (2009). Monocyte Chemoattractant Protein-1 (MCP-1): An Overview. J. Interf. Cytokine Res..

[23] Jin J., Lin J., Xu A., Lou J., Qian C., Li X., Wang Y., Yu W., Tao H. (2021). CCL2: An Important Mediator Between Tumor Cells and Host Cells in Tumor Microenvironment. Front. Oncol..

[24] Liu W., Wang L., Zhang J., Qiao L., Liu Y., Yang X., Zhang J., Zheng W., Ma Z. (2021). Purification of recombinant human chemokine CCL2 in E. coli and its function in ovarian cancer. 3 Biotech.

[25] Kuroda T., Kitadai Y., Tanaka S., Yang X., Mukaida N., Yoshihara M., Chayama K. (2005). Monocyte Chemoattractant Protein-1 Transfection Induces Angiogenesis and Tumorigenesis of Gastric Carcinoma in Nude Mice via Macrophage Recruitment. Clin. Cancer Res..

[26] Qian B.-Z., Li J., Zhang H., Kitamura T., Zhang J., Campion L.R., Kaiser E.A., Snyder L.A., Pollard J.W. (2011). CCL2 recruits inflammatory monocytes to facilitate breast-tumour metastasis. Nature.

[27] Xu W., Wei Q., Han M., Zhou B., Wang H., Zhang J., Wang Q., Sun J., Feng L., Wang S. (2018). CCL2-SQSTM1 positive feedback loop suppresses autophagy to promote chemoresistance in gastric cancer. Int. J. Biol. Sci..

[28] Lee G.T., Kwon S.J., Kim J., Kwon Y.S., Lee N., Hong J.H., Jamieson C., Kim W.-J., Kim I.Y. (2018). WNT5A induces castration-resistant prostate cancer via CCL2 and tumour-infiltrating macrophages. Br. J. Cancer.

[29] Feng H., Liu K., Shen X., Liang J., Wang C., Qiu W., Cheng X., Zhao R. (2020). Targeting tumor cell-derived CCL2 as a strategy to overcome Bevacizumab resistance in ETV5+ colorectal cancer. Cell Death Dis..

[30] Choi J., Lee H.J., Yoon S., Ryu H.-M., Lee E., Jo Y., Seo S., Kim D., Lee C.H., Kim W. (2020). Blockade of CCL2 expression overcomes intrinsic PD-1/PD-L1 inhibitor-resistance in transglutaminase 2-induced PD-L1 positive triple negative breast cancer. Am. J. Cancer Res..

[31] Tas F., Karabulut S., Serilmez M., Karabulut M., Duranyildiz D. (2015). Elevated circulating monocyte chemoattractant protein 1 (MCP-1/CCL-2) level may be an unfavorable predictive factor to platinum- and taxane-based combination chemotherapy in patients with gastric cancer. Cancer Chemother. Pharmacol..

[32] Natsagdorj A., Izumi K., Hiratsuka K., Machioka K., Iwamoto H., Naito R., Makino T., Kadomoto S., Shigehara K., Kadono Y. (2018). CCL2 induces resistance to the antiproliferative effect of cabazitaxel in prostate cancer cells. Cancer Sci..

[33] Qian D.Z., Rademacher B.L., Pittsenbarger J., Huang C.-Y., Myrthue A., Higano C.S., Garzotto M., Nelson P.S., Beer T.M. (2009). CCL2 is induced by chemotherapy and protects prostate cancer cells from docetaxel-induced cytotoxicity. Prostate.

[34] Wang T., Zhan Q., Peng X., Qiu Z., Zhao T. (2018). CCL2 influences the sensitivity of lung cancer A549 cells to docetaxel. Oncol. Lett..

[35] Moisan F., Francisco E.B., Brozovic A., Duran G.E., Wang Y.C., Chaturvedi S., Seetharam S., Snyder L.A., Doshi P., Sikic B.I. (2014). Enhancement of paclitaxel and carboplatin therapies by CCL2 blockade in ovarian cancers. Mol. Oncol..

[36] Takeyama Y., Kato M., Tamada S., Azuma Y., Shimizu Y., Iguchi T., Yamasaki T., Gi M., Wanibuchi H., Nakatani T. (2020). Myeloid-derived suppressor cells are essential partners for immune checkpoint inhibitors in the treatment of cisplatin-resistant bladder cancer. Cancer Lett..

[37] Qian Y., Ding P., Xu J., Nie X., Lu B. (2022). CCL2 activates AKT signaling to promote glycolysis and chemoresistance in glioma cells. Cell Biol. Int..

[38] Li D., Ji H., Niu X., Yin L., Wang Y., Gu Y., Wang J., Zhou X., Zhang H., Zhang Q. (2019). Tumor-associated macrophages secrete CC-chemokine ligand 2 and induce tamoxifen resistance by activating PI3K/Akt/mTOR in breast cancer. Cancer Sci..

[39] Izumi K., Fang L.Y., Mizokami A., Namiki M., Li L., Lin W.J., Chang C. (2013). Targeting the androgen receptor with siRNA promotes prostate cancer metastasis through enhanced macrophage recruitment via CCL2/CCR2-induced STAT3 activation. EMBO Mol. Med..

[40] Cho H.R., Kumari N., Vu H.T., Kim H., Park C.-K., Choi S.H. (2019). Increased Antiangiogenic Effect by Blocking CCL2-dependent Macrophages in a Rodent Glioblastoma Model: Correlation Study with Dynamic Susceptibility Contrast Perfusion MRI. Sci. Rep..

[41] Zhou S.-L., Zhou Z.-J., Hu Z.-Q., Huang X.-W., Wang Z., Chen E.-B., Fan J., Cao Y., Dai Z., Zhou J. (2016). Tumor-Associated Neutrophils Recruit Macrophages and T-Regulatory Cells to Promote Progression of Hepatocellular Carcinoma and Resistance to Sorafenib. Gastroenterology.

[42] Steinberg S.M., Shabaneh T.B., Zhang P., Martyanov V., Li Z., Malik B.T., Wood T.A., Boni A., Molodtsov A., Angeles C.V. (2017). Myeloid Cells That Impair Immunotherapy Are Restored in Melanomas with Acquired Resistance to BRAF Inhibitors. Cancer Res..

[43] Vergani E., Di Guardo L., Dugo M., Rigoletto S., Tragni G., Ruggeri R., Perrone F., Tamborini E., Gloghini A., Arienti F. (2015). Overcoming melanoma resistance to vemurafenib by targeting CCL2-induced miR-34a, miR-100 and miR-125b. Oncotarget.

[44] Sun W., Wang X., Wang D., Lu L., Lin H., Zhang Z., Jia Y., Nie X., Liu T., Fu W. (2022). CD40×HER2 bispecific antibody overcomes the CCL2-induced trastuzumab resistance in HER2-positive gastric cancer. J. Immunother. Cancer.

[45] Molon B., Ugel S., Del Pozzo F., Soldani C., Zilio S., Avella D., De Palma A., Mauri P., Monegal A., Rescigno M. (2011). Chemokine nitration prevents intratumoral infiltration of antigen-specific T cells. J. Exp. Med..

[46] Liu C., Yang Y., Chen C., Li L., Li J., Wang X., Chu Q., Qiu L., Ba Q., Li X. (2021). Environmental eustress modulates β-ARs/CCL2 axis to induce anti-tumor immunity and sensitize immunotherapy against liver cancer in mice. Nat. Commun..

[47] Rocha C.R.R., Silva M.M., Quinet A., Cabral-Neto J.B., Menck C.F.M. (2018). DNA repair pathways and cisplatin resistance: An intimate relationship. Clinics.

[48] Hato S.V., Khong A., de Vries I.J.M., Lesterhuis W.J. (2014). Molecular Pathways: The Immunogenic Effects of Platinum-Based Chemotherapeutics. Clin. Cancer Res..

[49] Shagufta, Ahmad I. (2018). Tamoxifen a pioneering drug: An update on the therapeutic potential of tamoxifen derivatives. Eur. J. Med. Chem..

[50] Shore N.D., Abrahamsson P.-A., Anderson J., Crawford E.D., Lange P. (2012). New considerations for ADT in advanced prostate cancer and the emerging role of GnRH antagonists. Prostate Cancer Prostatic Dis..

[51] Niu Y., Guo C., Wen S., Tian J., Luo J., Wang K., Tian H., Yeh S., Chang C. (2018). ADT with antiandrogens in prostate cancer induces adverse effect of increasing resistance, neuroendocrine differentiation and tumor metastasis. Cancer Lett..

[52] Wang X., Brea L., Lu X., Gritsina G., Park S.H., Xie W., Zhao J.C., Yu J. (2022). FOXA1 inhibits hypoxia programs through transcriptional repression of HIF1A. Oncogene.

[53] Apte R.S., Chen D.S., Ferrara N. (2019). VEGF in Signaling and Disease: Beyond Discovery and Development. Cell.

[54] Garcia J., Hurwitz H.I., Sandler A.B., Miles D., Coleman R.L., Deurloo R., Chinot O.L. (2020). Bevacizumab (Avastin®) in cancer treatment: A review of 15 years of clinical experience and future outlook. Cancer Treat. Rev..

[55] Colombo M. (2009). Sorafenib in Advanced Hepatocellular Carcinoma: A Further Step Toward Personalized Therapy of Liver Cancer. Gastroenterology.

[56] Menzer C., Menzies A.M., Carlino M.S., Reijers I., Groen E.J., Eigentler T., de Groot J.W.B., van der Veldt A.A., Johnson D.B., Meiss F. (2019). Targeted Therapy in Advanced Melanoma With Rare *BRAF* Mutations. J. Clin. Oncol..

[57] Erkes D.A., Cai W., Sanchez I.M., Purwin T.J., Rogers C., Field C.O., Berger A.C., Hartsough E.J., Rodeck U., Alnemri E.S. (2020). Mutant BRAF and MEK Inhibitors Regulate the Tumor Immune Microenvironment via Pyroptosis. Cancer Discov..

[58] Triulzi T., Forte L., Regondi V., Di Modica M., Ghirelli C., Carcangiu M.L., Sfondrini L., Balsari A., Tagliabue E. (2018). HER2 signaling regulates the tumor immune microenvironment and trastuzumab efficacy. OncoImmunology.

[59] Sharma P., Hu-Lieskovan S., Wargo J.A., Ribas A. (2017). Primary, Adaptive, and Acquired Resistance to Cancer Immunotherapy. Cell.

[60] Yang Y. (2015). Cancer immunotherapy: Harnessing the immune system to battle cancer. J. Clin. Investig..

[61] Garon E.B., Rizvi N.A., Hui R., Leighl N., Balmanoukian A.S., Eder J.P., Patnaik A., Aggarwal C., Gubens M., Horn L. (2015). Pembrolizumab for the Treatment of Non–Small-Cell Lung Cancer. N. Engl. J. Med..

[62] Meti N., Esfahani K., Johnson N.A. (2018). The Role of Immune Checkpoint Inhibitors in Classical Hodgkin Lymphoma. Cancers.

[63] Weber J.S., D’Angelo S.P., Minor D., Hodi F.S., Gutzmer R., Neyns B., Hoeller C., Khushalani N.I., Miller W.H., Lao C.D. (2015). Nivolumab versus chemotherapy in patients with advanced melanoma who progressed after anti-CTLA-4 treatment (CheckMate 037): A randomised, controlled, open-label, phase 3 trial. Lancet Oncol..

[64] Skoulidis F., Goldberg M.E., Greenawalt D.M., Hellmann M.D., Awad M.M., Gainor J.F., Schrock A.B., Hartmaier R.J., Trabucco S.E., Gay L. (2018). *STK11/LKB1* Mutations and PD-1 Inhibitor Resistance in *KRAS*-Mutant Lung Adenocarcinoma. Cancer Discov..

[65] Levina V., Su Y., Nolen B., Liu X., Gordin Y., Lee M., Lokshin A., Gorelik E. (2008). Chemotherapeutic drugs and human tumor cells cytokine network. Int. J. Cancer.

[66] Khurana A., Roy D., Kalogera E., Mondal S., Wen X., He X., Dowdy S., Shridhar V. (2015). Quinacrine promotes autophagic cell death and chemosensitivity in ovarian cancer and attenuates tumor growth. Oncotarget.

[67] Kirkin V., Lamark T., Sou Y.-S., Bjørkøy G., Nunn J.L., Bruun J.-A., Shvets E., McEwan D.G., Clausen T.H., Wild P. (2009). A Role for NBR1 in Autophagosomal Degradation of Ubiquitinated Substrates. Mol. Cell.

[68] Bartneck M., Schrammen P.L., Möckel D., Govaere O., Liepelt A., Krenkel O., Ergen C., McCain M.V., Eulberg D., Luedde T. (2019). The CCR2+ Macrophage Subset Promotes Pathogenic Angiogenesis for Tumor Vascularization in Fibrotic Livers. Cell. Mol. Gastroenterol. Hepatol..

[69] Koide N., Nishio A., Sato T., Sugiyama A., Miyagawa S.-I. (2004). Significance of macrophage chemoattractant protein-1 expression and macrophage infiltration in squamous cell carcinoma of the esophagus. Am. J. Gastroenterol..

[70] Salcedo R., Ponce M.L., Young H.A., Wasserman K., Ward J.M., Kleinman H.K., Oppenheim J.J., Murphy W.J. (2000). Human endothelial cells express CCR2 and respond to MCP-1: Direct role of MCP-1 in angiogenesis and tumor progression. Blood.

[71] Low-Marchelli J.M., Ardi V.C., Vizcarra E.A., van Rooijen N., Quigley J.P., Yang J. (2013). Twist1 Induces CCL2 and Recruits Macrophages to Promote Angiogenesis. Cancer Res..

[72] Shi C.-L., Yu C.-H., Zhang Y., Zhao D., Chang X.-H., Wang W.-H. (2011). Monocyte chemoattractant protein-1 modulates invasion and apoptosis of PC-3M prostate cancer cells via regulating expression of VEGF, MMP9 and caspase-3. Asian Pac. J. Cancer Prev..

[73] Fianco G., Mongiardi M.P., Levi A., De Luca T., Desideri M., Trisciuoglio D., Del Bufalo D., Cinà I., Di Benedetto A., Mottolese M. (2017). Caspase-8 contributes to angiogenesis and chemotherapy resistance in glioblastoma. eLife.

[74] Murdoch C., Muthana M., Coffelt S., Lewis C.E. (2008). The role of myeloid cells in the promotion of tumour angiogenesis. Nat. Cancer.

[75] Facciabene A., Peng X., Hagemann I.S., Balint K., Barchetti A., Wang L.-P., Gimotty P.A., Gilks C.B., Lal P., Zhang L. (2011). Tumour hypoxia promotes tolerance and angiogenesis via CCL28 and Treg cells. Nature.

[76] Li S., Lu J., Chen Y., Xiong N., Li L., Zhang J., Yang H., Wu C., Zeng H., Liu Y. (2017). MCP-1-induced ERK/GSK-3β/Snail signaling facilitates the epithelial–mesenchymal transition and promotes the migration of MCF-7 human breast carcinoma cells. Cell. Mol. Immunol..

[77] Lin T.-H., Lee S.O., Niu Y., Xu D., Liang L., Li L., Yeh S.-D., Fujimoto N., Yeh S., Chang C. (2013). Differential Androgen Deprivation Therapies with Anti-androgens Casodex/Bicalutamide or MDV3100/Enzalutamide versus Anti-androgen Receptor ASC-J9® Lead to Promotion versus Suppression of Prostate Cancer Metastasis. J. Biol. Chem..

[78] Lin T., Izumi K., Lee S., Lin W.-J., Yeh S., Chang C. (2013). Anti-androgen receptor ASC-J9 versus anti-androgens MDV3100 (Enzalutamide) or Casodex (Bicalutamide) leads to opposite effects on prostate cancer metastasis via differential modulation of macrophage infiltration and STAT3-CCL2 signaling. Cell Death Dis..

[79] Belli C., Trapani D., Viale G., D’Amico P., Duso B.A., Della Vigna P., Orsi F., Curigliano G. (2018). Targeting the microenvironment in solid tumors. Cancer Treat. Rev..

[80] Binnewies M., Roberts E.W., Kersten K., Chan V., Fearon D.F., Merad M., Coussens L.M., Gabrilovich D.I., Ostrand-Rosenberg S., Hedrick C.C. (2018). Understanding the tumor immune microenvironment (TIME) for effective therapy. Nat. Med..

[81] Funes S.C., Rios M., Escobar-Vera J., Kalergis A.M. (2018). Implications of macrophage polarization in autoimmunity. Immunology.

[82] Lin Y., Xu J., Lan H. (2019). Tumor-associated macrophages in tumor metastasis: Biological roles and clinical therapeutic applications. J. Hematol. Oncol..

[83] Mantovani A., Sozzani S., Locati M., Allavena P., Sica A. (2002). Macrophage polarization: Tumor-associated macrophages as a paradigm for polarized M2 mononuclear phagocytes. Trends Immunol..

[84] Liew P.X., Kubes P. (2019). The Neutrophil’s Role During Health and Disease. Physiol. Rev..

[85] Fujita S., Ikeda T. (2016). The CCL2-CCR2 Axis in Lymph Node Metastasis From Oral Squamous Cell Carcinoma: An Immunohistochemical Study. J. Oral Maxillofac. Surg..

[86] Lu H., Wu B., Ma G., Zheng D., Song R., Huang E., Mao M., Lu B. (2017). Melatonin represses oral squamous cell carcinoma metastasis by inhibiting tumor-associated neutrophils. Am. J. Transl. Res..

[87] Cheng Y., Li H., Deng Y., Tai Y., Zeng K., Zhang Y., Liu W., Zhang Q., Yang Y. (2018). Cancer-associated fibroblasts induce PDL1+ neutrophils through the IL6-STAT3 pathway that foster immune suppression in hepatocellular carcinoma. Cell Death Dis..

[88] Curiel T.J., Coukos G., Zou L., Alvarez X., Cheng P., Mottram P., Evdemon-Hogan M., Conejo-Garcia J.R., Zhang L., Burow M. (2004). Specific recruitment of regulatory T cells in ovarian carcinoma fosters immune privilege and predicts reduced survival. Nat. Med..

[89] Ge Y., Böhm H.-H., Rathinasamy A., Xydia M., Hu X., Pincha M., Umansky L., Breyer C., Hillier M., Bonertz A. (2019). Tumor-Specific Regulatory T Cells from the Bone Marrow Orchestrate Antitumor Immunity in Breast Cancer. Cancer Immunol. Res..

[90] Liu H., Wang S.-H., Chen S.-C., Chen C.-Y., Lin T.-M. (2019). Zoledronic acid blocks the interaction between breast cancer cells and regulatory T-cells. BMC Cancer.

[91] Mondini M., Loyher P.-L., Hamon P., de Thoré M.G., Laviron M., Berthelot K., Clémenson C., Salomon B.L., Combadière C., Deutsch E. (2019). CCR2-Dependent Recruitment of Tregs and Monocytes Following Radiotherapy Is Associated with TNFα-Mediated Resistance. Cancer Immunol. Res..

[92] Gabrilovich D.I., Nagaraj S. (2009). Myeloid-derived suppressor cells as regulators of the immune system. Nat. Rev. Immunol..

[93] Hartwig T., Montinaro A., von Karstedt S., Sevko A., Surinova S., Chakravarthy A., Taraborrelli L., Draber P., Lafont E., Vargas F.A. (2017). The TRAIL-Induced Cancer Secretome Promotes a Tumor-Supportive Immune Microenvironment via CCR2. Mol. Cell.

[94] Flores-Toro J.A., Luo D., Gopinath A., Sarkisian M.R., Campbell J.J., Charo I.F., Singh R., Schall T.J., Datta M., Jain R.K. (2020). CCR2 inhibition reduces tumor myeloid cells and unmasks a checkpoint inhibitor effect to slow progression of resistant murine gliomas. Proc. Natl. Acad. Sci. USA.

[95] Xiao F., Liu N., Ma X., Qin J., Liu Y., Wang X. (2020). M2 macrophages reduce the effect of gefitinib *by* activating AKT / mTOR in gefitinib-resistant cell lines HCC827 / GR. Thorac. Cancer.

[96] Arasada R.R., Shilo K., Yamada T., Zhang J., Yano S., Ghanem R., Wang W., Takeuchi S., Fukuda K., Katakami N. (2018). Notch3-dependent β-catenin signaling mediates EGFR TKI drug persistence in EGFR mutant NSCLC. Nat. Commun..

[97] Li X., Yao W., Yuan Y., Chen P., Li B., Li J., Chu R., Song H., Xie D., Jiang X. (2015). Targeting of tumour-infiltrating macrophages via CCL2/CCR2 signalling as a therapeutic strategy against hepatocellular carcinoma. Gut.

[98] Sanford D.E., Belt B.A., Panni R.Z., Mayer A., Deshpande A.D., Carpenter D., Mitchem J.B., Plambeck-Suess S.M., Worley L.A., Goetz B.D. (2013). Inflammatory Monocyte Mobilization Decreases Patient Survival in Pancreatic Cancer: A Role for Targeting the CCL2/CCR2 Axis. Clin. Cancer Res..

[99] Pienta K.J., Machiels J.-P., Schrijvers D., Alekseev B., Shkolnik M., Crabb S.J., Li S., Seetharam S., Puchalski T.A., Takimoto C. (2012). Phase 2 study of carlumab (CNTO 888), a human monoclonal antibody against CC-chemokine ligand 2 (CCL2), in metastatic castration-resistant prostate cancer. Investig. New Drugs.

[100] Nywening T.M., Wang-Gillam A., Sanford D., Belt B., Panni R.Z., Cusworth B.M., Toriola A.T., Nieman R.K., Worley L., Yano M. (2016). Targeting tumour-associated macrophages with CCR2 inhibition in combination with FOLFIRINOX in patients with borderline resectable and locally advanced pancreatic cancer: A single-centre, open-label, dose-finding, non-randomised, phase 1b trial. Lancet Oncol..

